# Histological artifacts induced by the contrast agent in Multi-Phase Post-Mortem CT Angiography (MPMCTA): Part I – Normal Tissues

**DOI:** 10.1007/s12024-025-01117-1

**Published:** 2025-11-15

**Authors:** Jessika Camatti, Bruno Giuliano Gangi, Maria Paola Bonasoni, Giovanni Battinelli, Luca Alemanno, Giovanni Pizzuti, Pietro Torricelli, Enrico Silingardi, Rossana Cecchi, Anna Laura Santunione

**Affiliations:** 1 University of Parma, Parma, Italy; 2 AUSL Modena, Modena, Italy; 3 AUSL Reggio Emilia, Reggio Emilia, Italy; 4 University Hospital Modena, Modena, Italy; 5 University of Modena and Reggio Emilia, Modena, Italy

**Keywords:** Post-mortem angiography, Multi-Phase Post-Mortem Computed Tomography Angiography, Forensic imaging, Post-mortem imaging, Histological changes, Histological artifacts

## Abstract

**Introduction:**

Multiphase postmortem computed tomographic angiography (MPMCTA) is a technique that provides comprehensive visualization of the cardiovascular system by injection of a contrast agent into the cadaver. Our aim is to systematically describe the histo-morphological changes and artifacts induced by the injection of the mixture of contrast agents (Angiofil® and paraffin oil) used for MPMCTA.

**Materials and methods:**

Histological specimens taken from 37 cases undergoing MPMCTA and autopsy were analyzed. Histological sections were analyzed using a Semi-motorized Olympus BX53 microscope associated with Olympus DP21 camera for images acquisition, in consecutive reading sessions by three forensic pathologists with experience in the field of histopathology for 20 years.

**Results:**

A total of 278 histological samples was included in the study. Different types of artifacts were encountered, which can be grouped into four main categories: the presence of optical empty spaces; the compression of surrounding structures; hyperaemia of small vessels and capillaries; vessel dilation.

**Conclusions:**

Postmortem angiography is a valuable method for visualizing blood vessels. However, the use of contrast media can introduce histological artifacts. This potential impact should be carefully considered when deciding whether to perform the procedure.

## Introduction

Postmortem imaging is now considered a significant adjunct to conventional autopsy, finding its value in both natural and traumatic deaths. Thus, post-mortem computed tomography (PMCT), post-mortem magnetic resonance (PMMR) and post-mortem angiography (PMA) have been increasingly developed and applied in recent years [1–4].

PMCT angiography (PMCTA) uses contrast medium to enhance image contrast between vessels and soft tissues, enabling detailed visualization of cardiovascular injuries [3]. Various techniques, contrast agents and carrier substances can be adopted. Oily substances of different viscosities were primarily used, but aqueous solutions and clinical contrast agents were also tested [5].

Injections of contrast agent can be made at various vascular sites—femoral, carotid, axillary, or subclavian one. Infusion methods include manual injection, though maintaining proper pressure is challenging, or using modified heart–lung machines, chest compressions, or pressure-controlled perfusion devices [6].

Multiphase post-mortem computed tomography angiography (MPMCTA) is a minimally invasive imaging technique that enhances autopsy findings by providing complete visualization of the cardiovascular system thanks to the injection of a contrast medium via a specifically designed pressure-controlled perfusione device (Virtangio®—Fumedica AG, Muri, Switzerland). Consequently, MPMCTA is a valuable tool for detecting, even in small vessels, haemorrhages, vessel ruptures, stenosis, aneurysms, and dissections, thereby enabling the detection of small intraparenchymal lesions [7, 8].

The main indications for MPMCTA include organ-specific analysis of vascular patterns, lesions, anatomical changes, and both pathological and physiological observations [1]. It is known that perfusing the corpse with a contrast agent—both oily and aqueous contrast agents—can cause histo-morphological artifacts [8–11]. Since all organs are perfused with contrast media during MPMCTA, such artifacts can potentially be observed in all samples collected for histological examination during autopsy. Recognizing and describing these artifacts is crucial to avoid misinterpretation, especially when histological investigations play a key role in determining the cause of death [8, 9, 11].

Little is known about the description of histo-morphological alterations and artifacts induced by MPMCTA, and most studies have been conducted on eviscerated organs after arterial and venous manual perfusion with a contrast medium [8, 10–12].

Our aim is to systematically describe the histo-morphological alterations in each organ induced by the injection in a corpse of a contrast agent mixture (Angiofil® and paraffin oil) used for MPMCTA.

## Materials and methods

### Casuistry

A retrospective study of the caseload of MPMCTA cases from the Legal Medicine Institute in Modena, Italy, between April 2016 and July 2019 was performed. This cohort consisted of 37 cases of sudden death. The post-mortem interval (PMI) of the included cases ranged from 22 to 110 h (mean: 55 h).

The authors declare that MPMCTA were performed for diagnostic purposes in the cases of unexpected death included in the study. Therefore, no informed consent could be requested. The autopsy samples collected for histological investigations to determine the cause of death were anonymized for the study. This report does not contain any personal information that could lead to the identification of the patients.

### Protocol

In each case, MPMCTA was performed according to the standardized protocol established by the Technical Working Group of Post Mortem Angiography Methods (TWGPAM; technical specifications available at: https://www.twgpam.org/). The imaging protocol comprised two native (unenhanced) scans followed by three contrast-enhanced angiographic phases. The examinations were conducted using a 64-detector CT scanner (TC LightSpeed VCT 64, Healthcare, Milwaukee, WI, USA) in conjunction with the Virtangio® device to infuse a contrast agent mixture of Angiofil® and paraffin oil. The slice thickness was 1.25 mm, with 0.75 mm collimation and a 512 × 512 matrix. All image reconstructions were performed on non-enhanced CT with a slice thickness of 2.0 mm, incremented by 1 mm, using soft tissue and bone kernels. The enhanced phases were reconstructed with a slice thickness of 1.0 mm, incremented by 0.5 mm, using the soft-tissue kernel.

Following radiological analysis, all bodies underwent external examination and autopsy following the ECLM recommendations [13]. Tissue sampling for histopathological evaluation was performed. Specimens were collected and formalin fixed, paraffin embedded and stained with Haematoxylin–Eosin (H&E) technique.

### Histo-morphological analysis

Histological sections were analysed using optical microscopy, using a Semi-motorized Olympus BX53 microscope associated with Olympus DP21 camera for images acquisition, in consecutive reading sessions by three forensic pathologists with experience in the field of histopathology for 20 years.

Exclusion criteria applied in this study comprised histological section which showed pathological processes, radiological signs of insufficient organ perfusion, insufficient histological staining, and severe autolytic changes.

## Results

A total of 494 histological samples were analyzed, with the sampling sites represented by cortex, basal nuclei, cerebellum, thyroid gland, thymus, lung, heart, vessel, liver, pancreas, spleen, kidney, adrenal gland, ovary, and prostate gland.

The present study included 270 of the 494 histological samples because the remaining 224 samples had features that met the exclusion criteria (insufficient organ perfusion: n = 13; pathological samples: n = 190; autolytic samples: n = 18; insufficient histological staining: n = 3). These data are detailed in Table 1.

**Table 1:** Samples analysed

*Organ*	*Number of samples analysed*	*Exclusion criterion: insufficient organ perfusion*	*Exclusion criterion: pathological* *samples*	*Exclusion criterion:* *autolytic samples*	*Exclusion criterion: insufficient histological staining*	*Number of samples excluded*	*Number of samples included in the study*
Cortex	27	8	7	0	0	15	**12**
Basal nuclei	7	2	0	0	0	2	**5**
Cerebellum	12	3	2	0	0	5	**7**
Thyroid gland	7	0	0	0	0	0	**7**
Thymus	3	0	0	0	0	0	**3**
Lung	76	0	18	0	0	18	**58**
Heart	146	0	54	0	0	54	**92**
Vessel	97	0	97	0	0	97	**0**
Liver	41	0	9	3	0	12	**29**
Pancreas	14	0	2	12	0	14	**0**
Spleen	22	0	0	0	1	1	**21**
Kidney	35	0	0	3	2	5	**30**
Adrenal gland	2	0	0	0	0	0	**2**
Ovary	3	0	1	0	0	1	**2**
Prostate gland	2	0	0	0	0	0	**2**
Total	494	13	190	18	3	224	**270**

Various types of artifacts were observed and can be classified into four main categories: the appearance of optical empty spaces (OES), compression of adjacent structures, hyperemia of small vessels and capillaries, and dilation of blood vessels. A systematic description of the histo-morphological changes and artifacts is now presented.

### Central nervous system

**Fig. 1:**
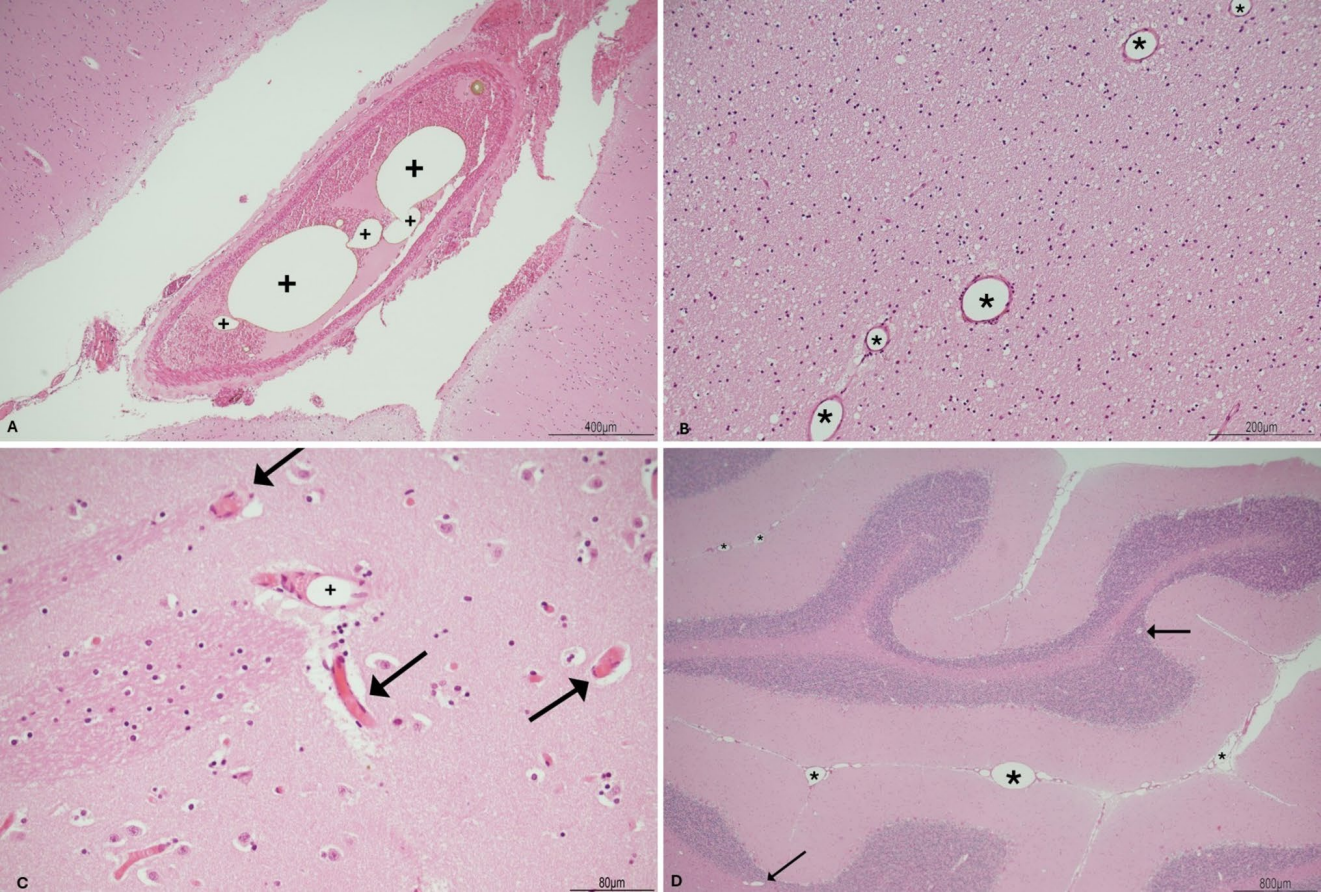
Central Nervous System. **a)** Cortex sample (4x), detail of a subarachnoid vessel with the presence of optical empty space (black +), which are characterized by the round shape and by the presence of chemical residues of the contrast medium. **b)** Cortex sample (10x), detail of perforating arterioles (black *) which appear dilated due to pressure induced by the contrast medium. **c)** Basal nuclei sample (20x), details of small hyperaemic vessels (black arrow) with the presence of optical empty spaces (black +). **d)** Cerebellum sample (2x), diffuse vascular dilation in both subarachnoid (black *) and intraparenchymal vessels (black arrow)

The central nervous system structures analyzed in this study, namely the brain cortex, cerebellum, and basal nuclei, were characterized by a small number of artifacts. The presence of OES was observed mostly in subarachnoid vessels, particularly in the larger ones, while they were rarely observed inside perforating arterioles and venules (Fig. 1a). The most frequent artifacts observed were the alternation of vessel dilation and hyperaemia (Fig. 1b, Fig. 1c, Fig. 1d).

### Cardiopulmonary system

**Fig. 2:**
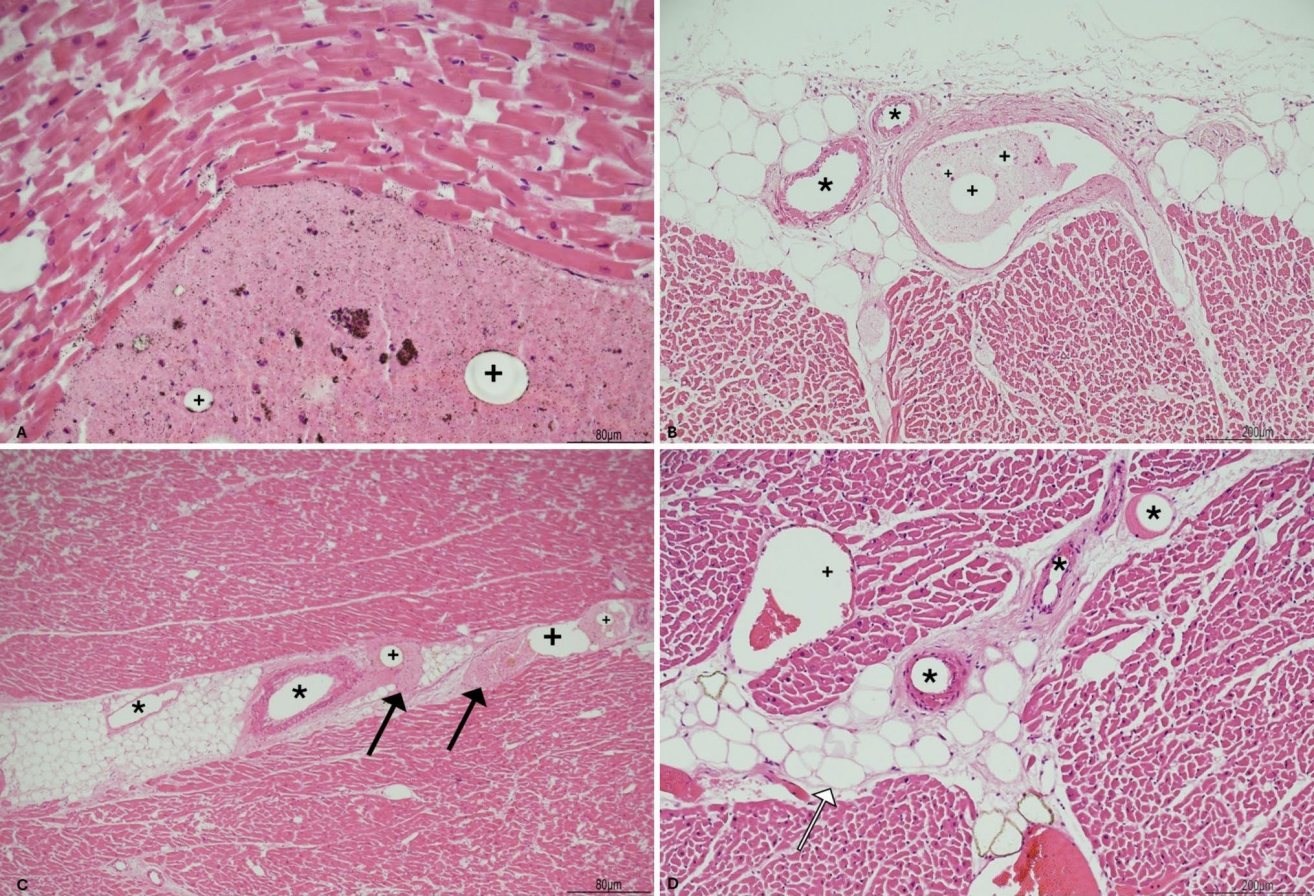
Myocardial tissue. **a)** Anterolateral heart wall (20x), detail of a septal vessel characterized by numerous optical empty spaces (black +). Please note that the black debris due to the chemical residue of the contrast medium is especially noticeable. **b)** Subepicardial vessel (10x), please note the presence of optical empty spaces in the arteriole (black +) and the venules dilation due to the presence of intravascular contrast medium (black *). **c)** Intramyocardial septa (4x), please note optical empty spaces presence (black +), vessel dilatation (black *) and hyperaemia (black arrow). **d)** Intramyocardial septa (10x), please note optical empty spaces (black +), which are located inside the vessel surrounded by erythrocytes, vessel dilation (black *) and adipocytes (white arrow), which are found outside the vessel, clearly demarked by the cytoplasmatic membrane

In the histological samples of the myocardium, a wide variety of patterns of permeation by the contrast medium were observed. In most cases, the epicardial segments of the coronary arteries appeared dilated and gaping, while the larger vessels in the septa were affected by all types of artifacts (Fig. 2a, Fig. 2b, Fig. 2c, Fig. 2d). The smaller vessels were almost exclusively affected by hyperaemia.

**Fig. 3:**
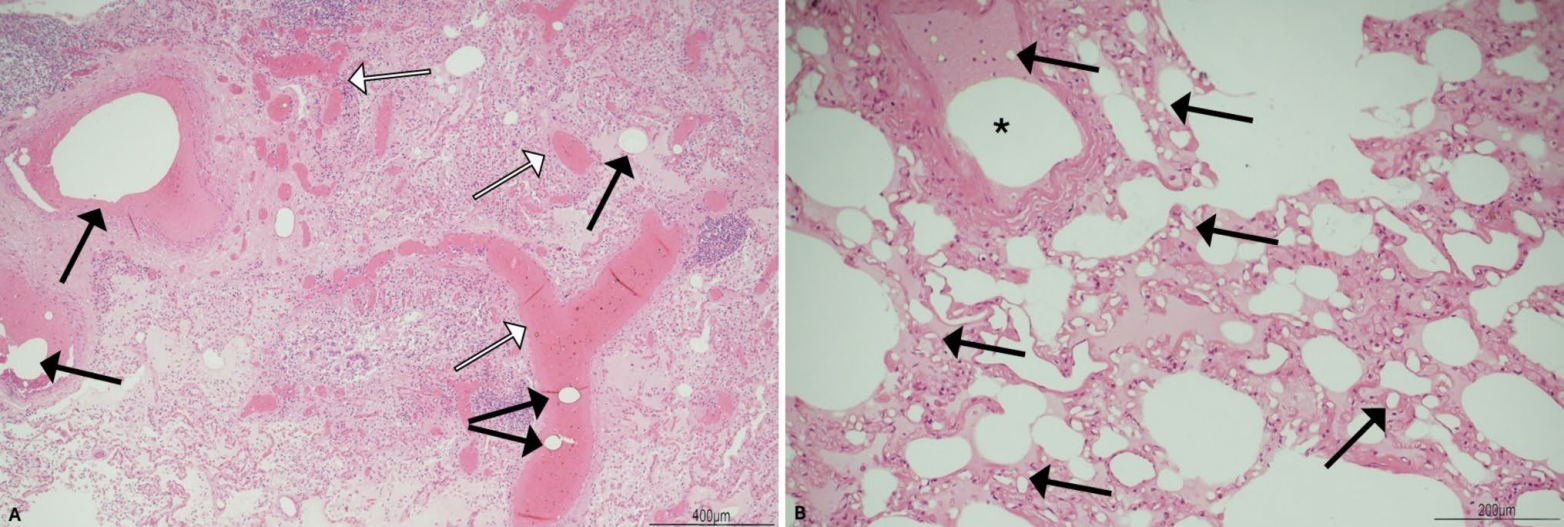
Lung tissue. **a)** Lung (4x), optical empty spaces (black arrow) of different dimensions inside the main vascular branch and diffuse hyperaemia (white arrow). **b)** Lung (10x), detail of the lobular and capillary system, please note diffuse hyperaemia, small optical empty spaces (black arrow) in the capillaries and bigger optical empty spaces in bigger vessels (black +)

In lung specimens, OES were frequently observed in the branches of the hilar and bronchial vessels (Fig. 3a, Fig. 3b). In the lobar and lobular branches, as well as in the capillary circulation, a pattern of widespread hyperaemia was observed.

### Genitourinary system

**Fig. 4:**
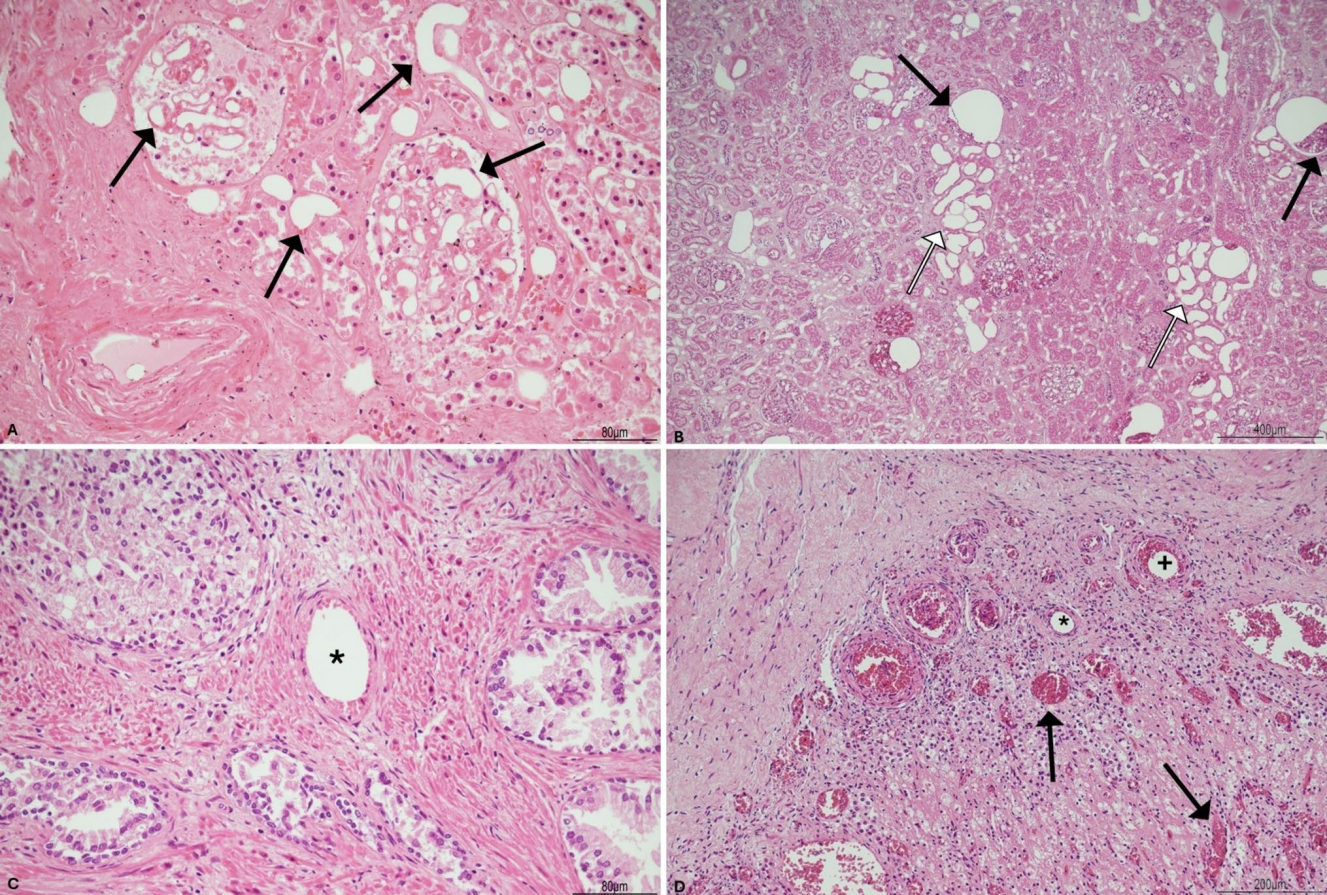
Genitourinary System. **a)** Kidney (20x), detail of the cortex, with the presence of dilation and optical empty spaces (black arrow) in the glomerular capillaries and in afferent arterioles. **b)** Kidney (4x), collapse of the glomerulus (black arrow) due to rupture of the vascular wall, with invasion of the renal tubules by the contrast medium (white arrow). **c)** Prostate (20x), vessel dilation (black *) with periferic erythrocytes. **d)** Ovary (10x), please note the presence of optical empty spaces (black +**)** beside vessel dilation (black *) and hyperaemia (black arrow)

In the kidney samples, the presence of dilation at the level of the glomerular capillaries, OES and dilation in the interlobular vessels, and hyperaemia at the level of the medullary rays were observed in almost all cases (Fig. 4a). In some samples, glomerular capillaries were so dilated that they altered the anatomical profile of the glomerulus, and, rarely, the collapse of the glomerulus itself due to rupture of the vascular wall, with invasion of the renal tubules by the contrast medium (Fig. 4b).

In all prostate gland histological preparation almost no artefacts were observed, probably due to the density of this organ which offers high resistance to contrast agent permeation (Fig. 4c).

In the ovary and fallopian tube samples, it was possible to observe the presence of OES in larger vessels and the alternation of dilation and hyperaemia in medium and small-caliber vessels (Fig. 4d).

### Other organs

**Fig.5:**
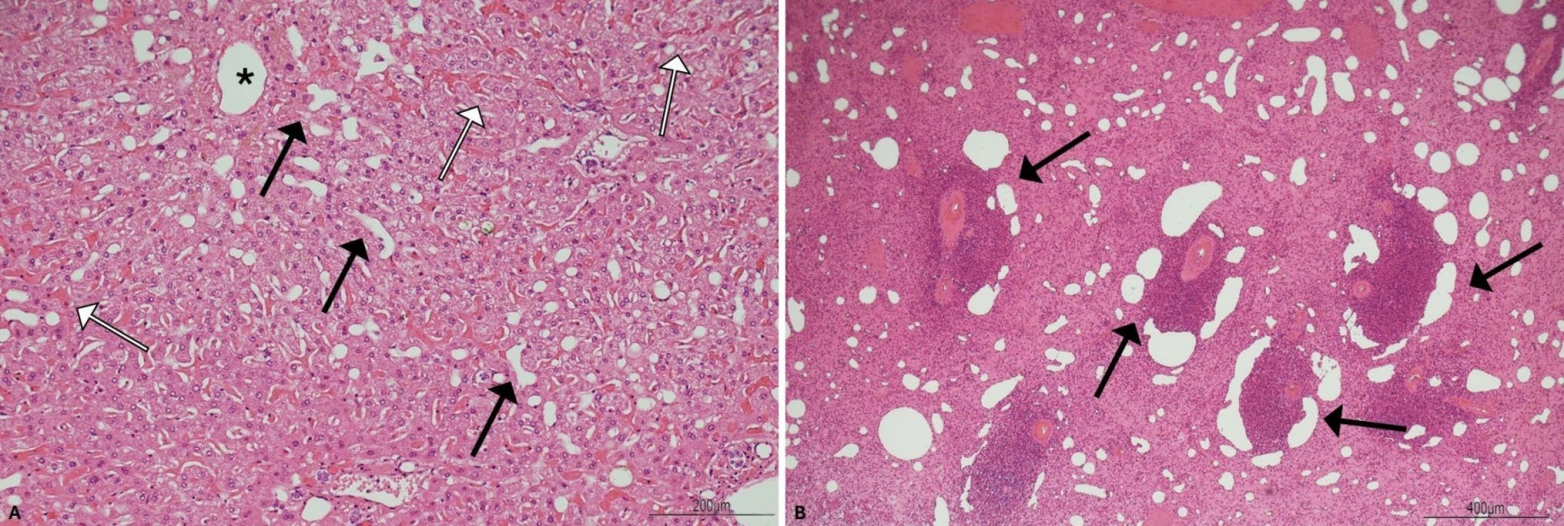
Other Organs. **a)** Liver (10x), detail of the lobule structure, where a dilated centrolobular venula (black *) and the alternation between dilated (black arrow) and hyperaemic (white arrow) capillaries can be observed. **b)** Spleen (4x), diffuse permeation of the contrast medium at the demarcation line between the splenic red and white pulp forming the typical “crown” image (black arrow)

In the liver, the most frequently observed artifact was vascular dilation, which generally involved all the vascular branches of the portal triad, extending to the sinusoids, which in many cases were also alternately affected by intense hyperaemia (Fig. 5a). In a few cases, the dilation of the sinusoids was so extensive that it caused compression of the hepatocyte plates, leading to their disorganization. The observation of OES was much less frequent and generally limited to medium-caliber branches.

The spleen samples presented a high number of artifacts. A recurring pattern that was observed was the presence of OES at the demarcation line between the splenic red and white pulp, forming a sort of 'crown' around the lymphatic tissue (Fig. 5b). Dilation of all vascular branches and the presence of OES within the splenic red pulp, with the white pulp being spared, were observed. In some cases, the presence of OES and the dilation of vascular spaces were so pronounced that they altered the tissue structure.

**Fig. 6:**
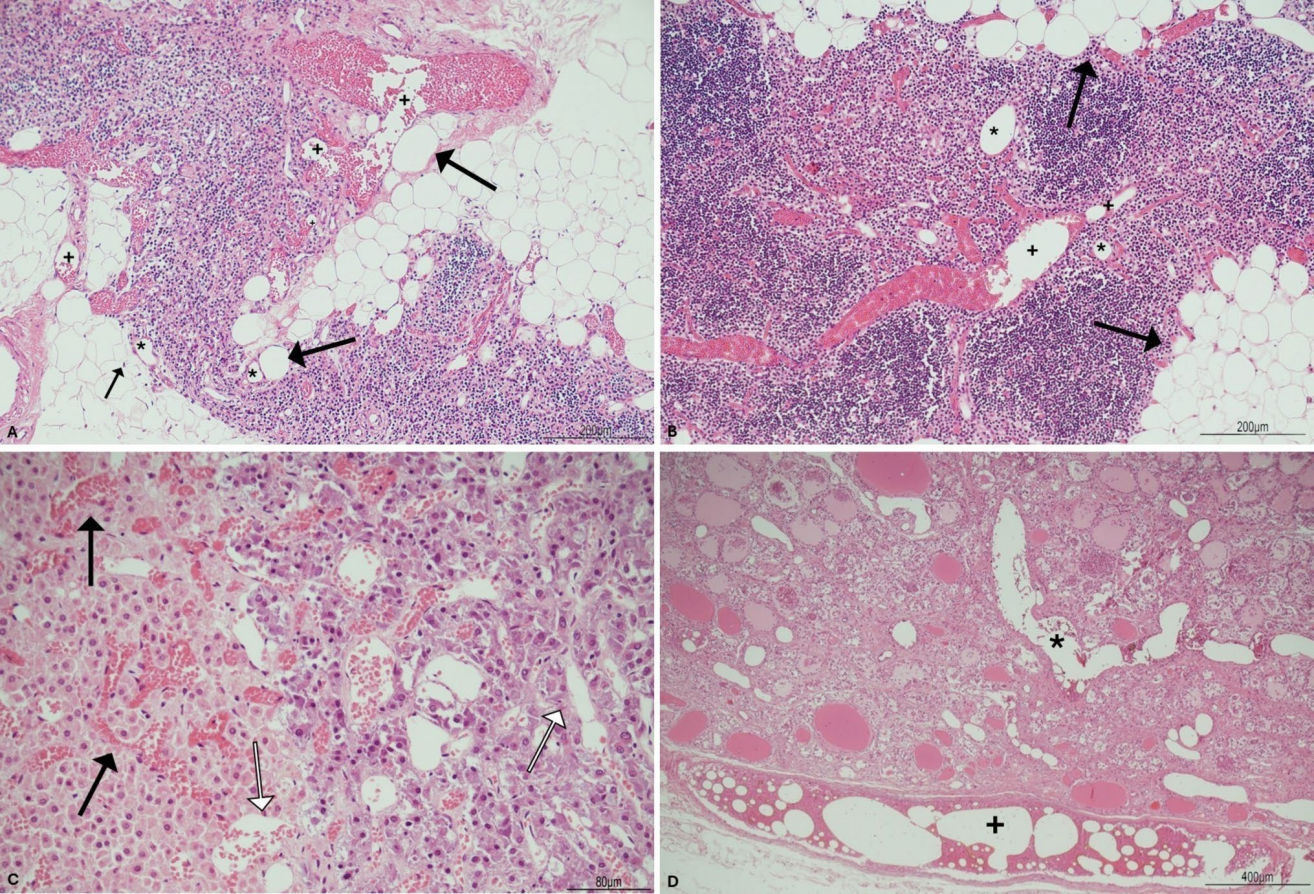
Other Organs.**a)** Thymus (10x), note the difference between optical empty spaces (black +), dilated vessels (black *) and adipocytes (black arrow). **b)** Thymus (10x), detail of a medium calibre vessel, please note optical empty spaces (black +), dilated vessels (black *) and adipocytes (black arrow). **c)** Adrenal Gland (20x), detail of the border between medulla and the zona reticularis, characterized by the alternation of hyperaemic (black arrow) and dilated vessel (white arrow). **d)** Thyroid Gland (4x), please note the presence of multiple optical empty spaces (black +) in bigger vessels and the dilation of medium calibre vessels (black *)

In the histological preparations of the thymus, the presence of OES was observed in medium-caliber vessels, while in small-caliber vessels and the capillary circulation, the alternation of dilation and hyperaemia prevailed (Fig. 6a, Fig. 6b).

In the adrenal gland, it was possible to observe involvement of the zona reticularis and of the medulla, with alternating dilated and hyperemic capillaries (Fig. 6c).

In the thyroid samples, it was possible to observe the presence of OES in large and medium-caliber vessels, dilation of the vascular wall in small-caliber vessels, and hyperaemia in the capillary circulation (Fig. 6d).

## Discussion

Forensic postmortem imaging plays a crucial role in modern forensic investigations by providing non-invasive, detailed visualization of the body after death. Postmortem imaging offers several significant advantages in forensic investigations, because it enables detailed three-dimensional visualization, which improves accuracy in identifying fractures, hemorrhages, or foreign objects [14–19].

Postmortem angiography is a valuable technique for visualizing the vascular tree in forensic investigations, allowing detailed assessment of blood vessels and detection of vascular injuries. By injecting a contrast medium, it makes it possible to visualize the entire circulatory system within the body, which aids in identifying causes of death related to vascular pathology. However, the effects of the contrast medium on surrounding tissues and its potential impact on subsequent histological analysis must be carefully considered. The contrast agents may alter tissue appearance or interfere with microscopic examination, so understanding these effects is essential to accurately interpret findings and avoid misdiagnosis during the forensic evaluation [9].

It is well known that the oily contrast agent can provide an image of fatty embolism in the lungs. Since MPMCTA with oil-based contrast medium leads to these false positive histological results, it should not be used to investigate such cases unless lung tissue samples have been previously collected – and, even in the latter case, the biopsy procedure itself could cause artefacts [20]. Some authors conclude that it is not possible to assess a potential fatal fatty embolism after MPMCTA with oily contrast agent. Therefore, MPMCTA should be performed with an aqueous contrast agent if there is any suspicion of fatty embolism [9].

Histo-morphological artifacts induced by contrast media used in MPMCTA have also been observed in the kidneys, where they can present as glomerular collapse. This artifact may significantly interfere with the interpretation of tissue morphology. Capuani et al*.* (2014) discussed a case in which a systemic vasculitis was diagnosed. In that case, the presence of glomerular collapse prevented a clear histological diagnosis, ultimately limiting the ability to better understand the renal involvement [11].

On the other hand, MPMCTA can significantly enhance the accuracy of histological sampling in forensic investigations. As Sabatasso et al*.* (2018) have pointed out, MPMCTA aids in identifying myocardial regions most likely to yield diagnostically valuable tissue samples. This targeted approach increases the probability of detecting myocardial infarction, if present, and thereby improves the chances of accurately determining the cause of death. In particular, areas of the myocardium that exhibit abnormal contrast enhancement should be prioritized for sampling, as these regions are more likely to harbor ischemic or necrotic changes indicative of infarction [21].

To the best of our knowledge, little is known about these artifacts, and most existing studies have been conducted in experimental settings.

The objective of this study is to systematically investigate potential artifacts associated with the oily contrast agent used in multi-phase postmortem computed tomography angiography (MPMCTA), specifically the mixture of Angiofil® and paraffin oil. Histological samples from 37 medico-legal autopsy cases, all of which underwent MPMCTA, were examined. Through detailed histopathological analysis, this study aims to identify and characterize any tissue alterations or artifacts attributable to the contrast agent. The findings are intended to support forensic pathologists in accurately interpreting histological changes following MPMCTA, thereby enhancing the diagnostic reliability of postmortem vascular imaging.

A total of 270 histological specimens were examined. In all cases, we were able to accurately identify the organ or tissue as well as the histological structures that characterize each tissue type. On the other hand, different types of artifacts were encountered, which can be grouped into four main categories. The first one refers to the OES, which result from the displacement of red blood cells within vessels by the oily medium and are the most common type of artifact, especially at the vascular level. OES often have curved/semilunar contours or a round shape, typically circumscribed by blackish material, which can be attributed to chemical residues from the contrast medium. The second one regards the compression of surrounding structures, which is common in organs with a loose vascular structure, where the distension of vessels filled with contrast medium exerts pressure on the surrounding parenchyma. The third type of artifacts relates to hyperaemia of small vessels and capillaries, which is due to the artificial propulsion of blood by the contrast solution under the pressure induced by the Virtangio®, giving the appearance of congestion. The fourth type of artifacts is associated to vessel dilation, which is also caused by the pressure induced by the Virtangio®, in which vessels filled with contrast medium appear completely empty, gaping, and with significantly distended walls.

Artifacts in the liver, particularly sinusoidal dilatation, are in differential diagnosis with the hepatic peliosis variant “parenchymal peliosis,” which consists of irregular cavities that are lined neither by sinusoidal cells nor by fibrous tissue, with the adjacent hepatic tissue occasionally showing necrosis of the hepatocytes [22]. The issue of differential diagnosis can play a role in the kidney as well, especially when collapsing glomerulopathy may be suspected, as the end-stage of focal segmental glomerulosclerosis [23]. Therefore, forensic pathologists should take this into account when examining specific autopsy samples.

Postmortem angiography is a powerful tool for visualizing the vascular system and enhancing the accuracy of forensic investigations. However, it is associated with the risk of inducing histological artifacts due to the use of contrast media. Because these artifacts can potentially interfere with microscopic examination and the interpretation of tissue samples, forensic pathologists should carefully consider, in advance, whether to perform postmortem angiography—especially in cases where detailed histological analysis is critical. This preliminary decision is essential to balance the benefits of vascular imaging against the possibility of compromising histological evaluation.

While the Atlas of Postmortem Angiography by Grabherr et al. (2016) [7] represents a landmark reference in the field by offering a descriptive and illustrative overview of angiographic findings, the present study addresses a different and complementary perspective. We provide a systematic histo-morphological assessment of artifacts induced by MPMCTA in a consecutive series of forensic autopsy cases. Unlike experimental or isolated organ studies, this approach allows the categorization of artifacts across multiple organ systems within a standardized medico-legal workflow, with quantitative data on sample inclusion and exclusion. To our knowledge, this is the first structured attempt to define the spectrum of histological artifacts in normal tissues following MPMCTA, thereby supporting forensic pathologists in differentiating imaging-related changes from genuine pathological findings during routine practice.

Our results clearly demonstrate that this method should not be performed in the presence of suspected fatty embolism, as the resulting images could be confused with artifacts. In contrast, marrow embolism remains identifiable, as does bone embolism [24]. Nevertheless, in cases where the cause of suspected death is attributed to the rupture of a major vessel, any potential artifacts do not compromise the interpretation of histological findings, since the diagnosis is primarily macroscopic, and microscopic images of the parenchyma are not relevant in such scenarios.

Further studies are needed to comprehensively characterize the artifacts induced by contrast media used in MPMCTA in pathological tissues. Pathological changes may alter tissue permeability, vascular integrity, or contrast retention, potentially resulting in misleading histological appearances. A deeper understanding of these interactions is essential to differentiate true pathological findings from imaging-related artifacts, ensuring accurate histopathological interpretation in post-mortem examinations.

Further research is needed to better identify and understand artifacts caused by contrast media in corpses at advanced stages of putrefaction. These situations may present additional challenges due to tissue degradation and altered vascular integrity, which can affect the appearance and interpretation of imaging and histological findings. Expanding studies to include such cases will help improve the accuracy and reliability of postmortem angiographic examinations across a wider range of forensic scenarios.

## Key points

- Multi-Phase Post-Mortem CT Angiography (MPMCTA) allows detailed visualization of the vascular system in forensic practice.
- The oily contrast mixture (Angiofil® with paraffin oil) induces characteristic histological artifacts.
- These artifacts mainly include optical empty spaces, vessel dilation, compression of adjacent structures, and hyperaemia.
- Recognizing such artifacts is essential to avoid misinterpretation in forensic histopathology.

## Data Availability

All relevant data are included in the manuscript.
